# Tenogenic differentiation protocol in xenogenic-free media enhances tendon-related marker expression in ASCs

**DOI:** 10.1371/journal.pone.0212192

**Published:** 2019-02-12

**Authors:** Deborah Stanco, Christian Caprara, Gianluca Ciardelli, Luca Mariotta, Mauro Gola, Greta Minonzio, Gianni Soldati

**Affiliations:** 1 Swiss Stem Cell Foundation, Gentilino, Switzerland; 2 Department of Mechanical and Aerospace Engineering, Politecnico di Torino, Turin, Italy; Università degli Studi della Campania, ITALY

## Abstract

Adipose-derived stem cells (ASCs) are multipotent and immune-privileged mesenchymal cells, making them ideal candidates for therapeutic purposes to manage tendon disorders. Providing safe and regulated cell therapy products to patients requires adherence to good manufacturing practices. To this aim we investigated the *in vitro* tenogenic differentiation potential of ASCs using a chemically defined serum-free medium (SF) or a xenogenic-free human pooled platelet lysate medium (hPL) suitable for cell therapy and both supplemented with CTGF, TGFβ-3, BMP-12 and ascorbic acid (AA) soluble factors. Human ASCs were isolated from 4 healthy donors and they were inducted to differentiate until 14 days in both hPL and SF tenogenic media (hPL-TENO and SF-TENO). Cell viability and immunophenotype profile were analysed to evaluate mesenchymal stem cell (MSC) characteristics in both xenogenic-free media. Moreover, the expression of stemness and tendon-related markers upon cell differentiation by RT-PCR, protein staining and cytofluorimetric analysis were also performed. Our results showed the two xenogenic-free media well support cell viability of ASCs and maintain their MSC nature as demonstrated by their typical immunophenototype profile and by the expression of NANOG, OCT4 and Ki67 genes. Moreover, both hPL-TENO and SF-TENO expressed significant high levels of the tendon-related genes SCX, COL1A1, COL3A1, COMP, MMP3 and MMP13 already at early time points in comparison to the respective controls. Significant up-regulations in scleraxis, collagen and tenomodulin proteins were also demonstrated at in both differentiated SF and hPL ASCs. In conclusion, we demonstrated firstly the feasibility of both serum and xenogenic-free media tested to culture ASCs moving forward the GMP-compliant approaches for clinical scale expansion of human MSCs needed for therapeutical application of stem cells. Moreover, a combination of CTGF, BMP-12, TGFβ3 and AA factors strongly and rapidly induce human ASCs to differentiate into tenocyte-like cells.

## Introduction

Tendons are ubiquitous, dense fibrous connective tissue made up primarily of collagenous fibers, with the essential role of transmitting contractile forces from muscle to the bone making movement of the body possible. Healing process in tendons occurs slowly and often leads to the formation of a tissue with inferior mechanical properties and high risk of reinjure. Current conservative and surgical treatments are still mainly symptomatic without providing a successful long-term solution as well as complete strength and functional recovery of the restored tendon. The urgent need for an advanced therapeutic that addresses the underlying pathology by improving clinical, mechanical, and radiologic outcomes is evident. However, although their high social impact and clinical significance, tendon biology and related injury mechanisms are currently poorly understood thus representing a limit to the therapeutic progress in this field [1, 2].

Tendon tissue engineering and stem cell-based therapy have been recognized as promising approaches to augment tendon repair by enhancing regeneration and restoring the functionality and characteristics that more closely resembles the native uninjured tissue [3,4]. Stem cells derived from adipose tissue (ASCs) represent the more abundant mesenchymal stem cell (MSC) source harvested using minimally invasive techniques, and can be produced according to current Good Manufacturing Practice (GMP) guidelines when not directly selected in the operating theatre. Cultured ASCs exhibit differentiative potential toward several cell lineages, as well as possess immunomodulatory properties, the ability to express anti-inflammatory cytokines and to prolongate allotransplant survival [5–10]. These favorable regenerative and paracrine abilities make ASCs currently under investigation for a high number of clinical therapeutic applications even if compared to bone- and cartilage-related pathologies, the use of MSCs in tendon related disorders has been investigated very little, so far [11–15]. Moreover, several efforts have been made to trigger in vitro MSC tenogenic differentiation using different types and concentrations of growth factors. However, there is still a limited consensus in literature about the best protocol and formulation to use also due to the scarce knowledge in tendon biology and therefore of tendon-related markers [16–20]. Furthermore, cell-based therapies must abide to the U.S. Food and Drug Administration (FDA) strict guidelines concerning the use of xenoproducts to provide a safe and regulated cell therapy product to patients [21]. The majority of studies were conducted using cultured ASCs in fetal bovine serum (FBS) that it traditionally employed to support cell growth and attachment. However, it is known that the use of FBS can exert a factitious cell response as well as an immune reaction being associated with pathogenic contamination and increase of immunogenicity of the cells [22, 23]. Studies concerning the standardization of procedures and GMP protocols to make the clinical use of stem cells possible with the development of safe-for-human-use materials have been addressed [23–26]. Although the common alternatives of the use of FBS for clinical-scale MSC expansion are human serum and platelet-derived products, the use of human serum may also include others concerns about safety and lot-to-lot variability issues [25, 26]. Thus, an important scientific and technological goal that must be achieved is the development of an ideal culture system suitable for cellular therapy represented by xenogenic- and serum-free medium with a chemically defined composition. Based on these purposes, the aim of this study was to evaluate for the first time the tenogenic differentiation potential of ASCs using a defined serum free medium (SF) or a xenogenic-free medium supplemented with human platelet lysate (hPL). The SF medium consisting of a blend of essential amino acids, inorganic salts, and other components, along with an optimized mix of the recombinant human growth factors already known to be essential for MSC expansion (PCT/EP2013/072738). The second medium here used was a commercial hPL supplemented medium, obtained by pooling more than 300 donors to contain the intrinsic donor variability. Cell morphology, immunophenotype, cell viability and expression of proliferative (Ki67 and PCNA) and stem-cell (KLF4, OCT4 and NANOG) markers were investigated in both hPL-ASCs and SF-ASCs. Therefore, ASCs were TENO-induce using for the first time a cocktail of ascorbic acid and CTGF, TGFβ-3, BMP-12 supplementation for 1, 3, 7 and 14 days and the tenogenic differentiation evaluated by quantifying the expression of the tendon‐related markers scleraxis (SCX), tenomodulin (TNMD), tenascin (TNC), cartilage oligomeric matrix protein (COMP), metalloproteinases (MMP3 and MMP13), tissue inhibitor of metalloproteinase (TIMP-2) and the ability of cells to form tendon‐like extracellular matrix. Finally, to better mimic the tendon tissue environment all cultures were conducted in flask surfaces coated with collagen type I since this is the major tendon ECM component.

## Materials and methods

This study was reviewed and approved by the board of the Swiss Stem Cell Foundation.

### ASC isolation and culture

Subcutaneous adipose tissue was collected from healthy human donors (n = 4) undergoing a liposuction and ASCs isolated, expanded and characterized according to the standard operating procedures developed by SSCF and already GMP compliant. All donors signed an informed consent declaration. Adipose tissue samples were collected, anonymized and sent to our research biobank facility as approved by the Ethical Committee of the Canton Ticino, Switzerland (CE 2961). The isolation of the stromal vascular fraction (SVF) was performed by a protocol developed in our laboratories (Patent PCT/EP2012/069261). Briefly, 150 ml of adipose tissue was washed twice with Dulbecco’s PBS (DPBS with Ca^2+^ and Mg^2+^, Gibco, Life Technologies, Oregon, USA) in a 100 ml syringe (BBraun Medical AG, Melsungen, Germany) and held vertically in a support stand for few minutes to spontaneously separate adipose tissue and hydrophilic fluids. Aqueous phase was discarded, and adipose tissue was digested with Liberase MNP-S (Roche Applied Science, Basel, Switzerland) at a final concentration of 0.28 Wünsch U/ml diluted in DPBS (with Ca^2+^ and Mg^2+^) and incubated at 37°C for 45 minutes under agitation. Enzymatic reaction was stopped by DPBS (without Ca^2+^ and Mg^2+^, Gibco, Life Technologies, Oregon, USA) supplemented with 1% albumin (CSL Behring AG, Bern, Switzerland) and strongly agitated to separate the hydrophilic phase from the hydrophobic one. The lower layer, which contains the SVF cells, was carefully poured out into a conical 50 ml centrifuge tube (Falcon, Corning Science, México) and washed with 1% albumin solution to increase cell yield. Finally, after filtration through a 100 μm and a 40 μm sieve (BD Falcon, Basel, Switzerland), the SVF was centrifuged at 400 g at room temperature for 5 minutes and cells were resuspended in 5% human albumin solution (CSL Behring AG, Switzerland). The SVF was then analyzed for cell count and viability using an automated propidium iodide-based cell counting device (Nucleocounter NC-100, Chemometec A/S, Denmark). For cryopreservation, SVF cells were centrifuged 5 minutes at 400 g, resuspended in an ice-cold solution of 1% albumin solution, 5.5% ME_2_SO and 4.5% dextran-40 (Cryosure DEX-40, WAK-Chemie Medical GmbH, Germany) in MEM alpha (PAA Laboratories, Austria) and transferred into a 2 ml cryovial (Nalgene, Thermo Fisher Scientific, Waltham, USA). Cells were frozen by means of a programmable freezer (Consartic GmbH, Germany) under the following conditions: from 4°C to 0°C in 6 minutes, then hold for 15 minutes at 0°C. From 0°C to -2°C in 9 minutes and then hold at -2°C for 2 minutes. From -2°C to -35°C in 25.5 minutes and finally, from -35°C to -100°C in 13 minutes. Cryovials were then transferred into liquid nitrogen for long-term storage.

### Cell culture

After thawing, SVF cells were culture for cell expansion in flask (Nunc, Thermo Fisher Scientific, USA) at 3x10^3^ cells/cm^2^ as previously described [27]. The cell expansion medium consisted of 5% pooled human platelet lysate (hPL, Stemulate, Cook Regentec, USA) in α MEM medium without nucleosides with Glutamax (Fisher Scientific, USA) supplemented of 0.1 μg Primocin (InvivoGen, USA). Fresh medium was supplied every 3 days. After approximately 7 days of culture, cells reached 80–90% confluence and the harvest was performed by TrypLE Select (Gibco). Counting and assessing of cell viability was conducted with an image cytometer based on fluorescence from the fluorescent dye, propidium iodide (PI) (Nucleocounter NC-100, ChemoMetec A/S, Denmark). In addition to the total count of cells in the sample the determination of viable cells is based on the “PI-exclusion” method. After 3 passages in culture ASCs were plated on pre-coated collagen I culture flasks (Corning, USA) at 3x10^3^ cells/cm^2^ of density in low percentage of hPL medium (1% hPL, Stemulate, Cook Regentec) or in the serum free medium (SF) patented by SSCF (PCT/EP2013/072738). A low percentage of hPL supplementation was chosen accordingly to manufacture instructions in order to obtain comparable concentrations of growth factors and soluble molecules between the two media (data not shown). The formulation of SF medium consists in Ham’s F12/IMDM (1:1) medium supplemented with 0.1 μg/ml Primocin, 2 mM L-alanyl-L-glutamine, 50 μg/ml L ascorbic acid-2-phosphate (Sigma Aldrich), 5 μg/ml human ITS supplement premix (BD Life Sciences), 250 μg/ml human Albumin 5% solution (CSL Behring AG), 50 ng/ml human thyroglobulin (Millipore SAS, Calbiochem, USA), and 10 ng/ml of the growth factors b-FGF, PDGF-AB, PDGF-BB, TGF-β1 (all from ProSpec-Tany TechnoGene Ltd, Israel). hPL-ASCs and SF-ASCs were detached and then analyzed for cell count and viability as show before.

### Tenogenic differentiation

Both hPL-ASC and SF-ASC populations at passage 4 were seeded at cell density of 3x10^3^ cells/cm^2^ in collagen I coated -well plate or culture flask (Corning, USA) and then induced to differentiate towards tenogenic lineage by culturing in hPL (hPL-TENO) or SF (SF-TENO) medium both supplemented with 50 μg/ml Ascorbic acid (AA; Sigma Aldrich), 50 ng/ml BMP-12, 100 ng/ml CTGF and 10 ng/ml TGF-β3 (all from PeptroTech, UK). Media were changed twice a week. Cells cultured in hPL or SF medium without any further supplementation were used as control (hPL-CTRL, SF-CTRL).

### Immunophenotyping by flow cytometry

SVF characterization was performed as previously described [28]. Briefly, after SVF isolation, 5x10^5^ cells were stained with the following monoclonal antibodies: CD146-PE, CD34-APC-A750, CD45-KrO (Beckman Coulter, Switzerland), Syto 40 (Life Technologies, USA) to exclude cellular debris and 7- amino-actinomycin D (7-AAD, Beckman Coulter) to assess cell viability. After 20 minutes of incubation, erythrocytes were lysed using Versalyse lysing solution (Beckman Coulter) and, before acquisition, Flow-Count Fluorospheres (Beckman Coulter) were added, to directly determine the absolute number of ASCs. ASCs were identified as the CD45 and CD146 negative and CD34 positive fraction [28, 29].

For immunophenotypic characterization of cultured hPL-ASCs and SF-ASCs, 5×10^5^ cells at passage 4 were incubated with LIVE/DEAD fixable stain (Life Technologies) at room temperature in the dark for 30 minutes. Cells were then stained with control antibodies IgG1- FITC, IgG3-PE, IgG1-PC5, IgG1-PC7, IgG1-APC, IgG1-APC-A750, IgG1-KrO (all from Beckman Coulter) or with CD73-FITC, CD31-FITC (BD Biosciences, USA), CD105-PE, CD90-PC5, CD13-PC7, CD44-APC-A750, CD45-KrO (Beckman Coulter). Before measurement, cells were resuspended in IOTest 3 Fixative Solution (Beckman Coulter). To evaluate tenomodulin (TNMD) expression after 7 and 14 days of differentiation, 2.5x10^5^ CTRL and TENO cells were incubated with anti-human polyclonal tenomodulin antibody (ab203676, Abcam) for 1 hour at 4°C at the dark. After incubation, goat polyclonal antibody Alexa Fluor 488 (ab150077, Abcam) and was used as secondary antibody. The false positive fluorescence emission by death cells was excluded by DAPI viability dye (Beckman Coulter) staining. All flow cytometry analyses were performed using a Navios 3-lasers/10-channels flow cytometer (Beckman Coulter), and data were analyzed with Kaluza software (Beckman Coulter).

### Cell viability

Undifferentiated and differentiated hPL-ASCs and SF-ASCs were harvest after 1, 7 and 14 days of culture and then cell counting and assessing of viable cells were performed with the automated propidium iodide-based cell counting device (Nucleocounter NC-100, ChemoMetec A/S). Moreover, cell viability was assessed in undifferentiated and differentiated hPL-ASCs and SF-ASCs by Alamar Blue Assay (Thermo Fisher Scientific, USA) at 1, 4 and 7 days of culture [30]. At the day of the evaluation cells were incubated with Alamar Blue (1:10 diluition in MEM) at 37°C in the dark. Four hours later, supernatants were transferred to black-bottom 96-well plates and emitted fluorescence was read with a Wallac Victor II plate reader (Perkin Elmer, Milan, Italy).

### RNA isolation and reverse transcription

At 1, 3, 7 and 14 days of differentiation, total RNA was extracted using the RNeasy Mini Kit (Qiagen, Hilden, Germany) according to manufacturer’s instruction. The concentration of extracted RNA from each sample was determined by spectrophotometric analysis with a Jenway Genova spectrophotometer (Bibby Scientific Limited, United Kingdom). cDNA was synthesized using Maxima H Minus First Strand cDNA Synthesis Kit (Thermo Scientific, USA) with oligodT and random hexamer primers according to manufacturer’s instruction. After reverse transcription, all cDNA samples were diluted to a final concentration of 5 ng/μl and stored at -80°C until use.

### Quantitative real-time polymerase chain reaction

Quantitative real-time polymerase chain reaction (qPCR) analysis was performed by SYBR Green technology. Reactions were set up for 10 ng of cDNA in a final volume of 20 μl in a 96-well plate (Bio-Rad, Hercules, USA) and processed in a CFX Connect Real Time PCR Detection System (Bio-Rad, Hercules, USA). The PCR master mix used contained 1 x SsoAdvanced SYBR Green Supermix (Bio-Rad, Hercules, USA), 250 nM forward primer, 250 nM reverse primer, up to 20 μl with nuclease-free water. Primers were designed by primer-BLAST (NCBI, USA), within the sequences of a panel of genes (SCX, TNC, DCN, COMP, COL1A1, COL3A1, MMP3, MMP13, TIMP2, KLF4, NANOG, Ki67, PCNA*)*, on an exon-exon junction in order to prevent genomic DNA amplification. To analyze the relative expression of different genes, three housekeeping genes were chosen (GAPDH, GUSB and YWHAZ) and the geometric mean of their Ct values was calculated [31]. A sample without cDNA was used to verify the absence of nucleic acid contaminations. A cDNA sample composed of a mix of various cell extracts was run of every 96-well plate and was then used as calibrator (CAL) to which the expression level of every gene was normalized. Thermocycler program consisted of an initial hot start cycle at 95°C for 30 seconds followed by 45 amplification cycles resulting in a denaturation step at 95°C for 10 seconds and an annealing-extension phase at 60°C for 30 seconds. For melt-curve evaluation at the end of the analysis, the temperature was raised from 65°C to 95°C at rate of 0.5°C every 5 seconds. For all samples, reactions were performed in duplicate. The Ct values were recorded with a threshold of 3000 relative fluorescence units and the relative gene expression, expressed as 2^-ΔCt^. Results are expressed as mean ± SD relative to CAL expression. Primers used in this work are reported in Table 1.

**Table 1:** Primers used in this study.

Gene Name	Sequence 5’-3’	Type	Amplicon length (bp)	Accession number
***GAPDH***	5:TTCGTCATGGGTGTGAACCA	housekeeping	142	NM_002046.3
	3:CTGTGGTCATGATGAGTCCTTCCA			
***GUSB***	5:CTCATTTGGAATTTTGCCGAATTTTT	housekeeping	81	NM_000181.3
	3:CCGAGTGAAGATCCCCTTTTTA			
***YWHAZ***	5:TGGCTCGAGAATACAGAGAG	housekeeping	99	NM_001135699
	3:GTGAAGCATTGGGGATCAAG			
***SCX***	5: CAGCGGCACACGGCGAAC	Tendon	163	BK000280
	3: CGTTGCCCAGGTGCGAGATG			
***TNC***	5:CCACAATGGCAGATCCTTCT	Tendon	118	NM_002160
	3: GTTAACGCCCTGACTGTGGT			
***DCN***	5:CTCTGCTGTTGACAATGGCTCTCT	Tendon	256	NM_001920
	3:TGGATGGCTGTATCTCCCAGTACT			
***COMP***	5:AGAAGTCCTATCGTTGGTTCC	Tendon	104	NM_000095
	3: CAAGACCACGTTGCTGTC			
***COL1A1***	5:CCAGAAGAACTGGTACATCAGCAA	Tendon	70	NM_000088.3
	3: CGCCATACTCGAACTGGAATC			
***COL3A1***	5:GGGAACATCCTCCTTCAACA	Tendon	183	NM_000090.3
	3:GCAGGGAACAACTTGATGGT			
***MMP3***	5:CTGTTGATTCTGCTGTTGAG	Tendon	126	NM_002422.4
	3:AAGTCTCCATGTTCTCTAACTG			
***MMP13***	5: AAGACTTCCCAGGAATTGGTGA	Tendon	126	NM_002427.4
	3: GGCATGACGCGAACAATACG			
***TIMP2***	5: ATCTCATTGCAGGAAAGGCCG	Tendon	103	NM_003255.4
	3: AGGCTCTTCTTCTGGGTGGT			
***KLF4***	5: AAGAGTTCCCATCTCAAGGCACA	Stemness	90	NM_001314052.1
	3: GGGCGAATTTCCATCCACAG			
***NANOG***	5: CAACTGGCCGAAGAATAGCAATG	Stemness	110	NM_001297698.1
	3: TGGTTGCTCCAGGTTGAATTGTT			
***KI67***	5: AGCAAGCACTTTGGAGAGCA	Proliferation	89	NM_001145966.1
	3: CATTGTCCTCAGCCTTCTTTGG			
***PCNA***	5: GTAGTAAAGATGCCTTCTGGTG	Proliferation	189	NM_002592.2
	3: TCTCTATGGTAACAGCTTCCTC			

Abbreviations: F, Forward Primer; R, Reverse Primer

### Immunofluorescence staining

Expression of the transcription factor scleraxis was assessed by immunofluorescence staining in both hPL and SF passage 4 ASCs cultured at 3x10^3^ of cell density on 22mm pre-coated collagen I German Glass coverslip (Corning) and induced toward tenogenic lineage as described before. After 3 days of differentiation, cells were fixed with 4% paraformaldehyde in PBS. Cells were washed 3 times in PBS, permeabilized with 0.5% Triton X-100 in PBS (PBST) and blocked with BSA (Sigma Aldrich). Immunostaining was performed overnight at 4°C using 1:100 goat anti-human Scleraxis (sc-87425, Santa Cruz Biotechnology). Cells were washed 3 times in PBST, incubated for 1 hour at room temperature with 1:1000 Alexa Fluor 488 rabbit anti-goat IgG (Invitrogen) and cell nuclei were counterstained with DAPI (BDBioscence). Immunostained cells were observed and photographed under a fluorescence microscope (Zeiss Axiophot).

### Sirius red staining

After 7 days of differentiation the total collagen deposition was evaluated in CTRL and TENO ASCs of both SF and HPL medium seeded in collagen I coated 24-well plate (Corning) at cell density of 3x10^3^ cells/cm^2^. Briefly each sample was fixed in Bouin’s solution (Bouin’s Fixative, Electron Microscopy Sciences, USA) for 1 hour and collagen fibers stained with 0.1% Sirius Red saturated in picric acid (Sigma). The collagen matrix deposition was finally visualized under polarized light microscopy [32].

### Statistical analysis

Data are expressed as means ± standard deviations. The normal distribution of values was assessed by the Kolmogorov–Smirnov normality test. Statistical analyses were performed using the Student’s t-test for data with a normal distribution and the Wilcoxon test for data with a non-normal distribution (GraphPad Prism v7.00; GraphPad Software, USA).

## Results

### Human ASCs maintain the typical stem cell features in hPL and SF media

**Fig 1:**
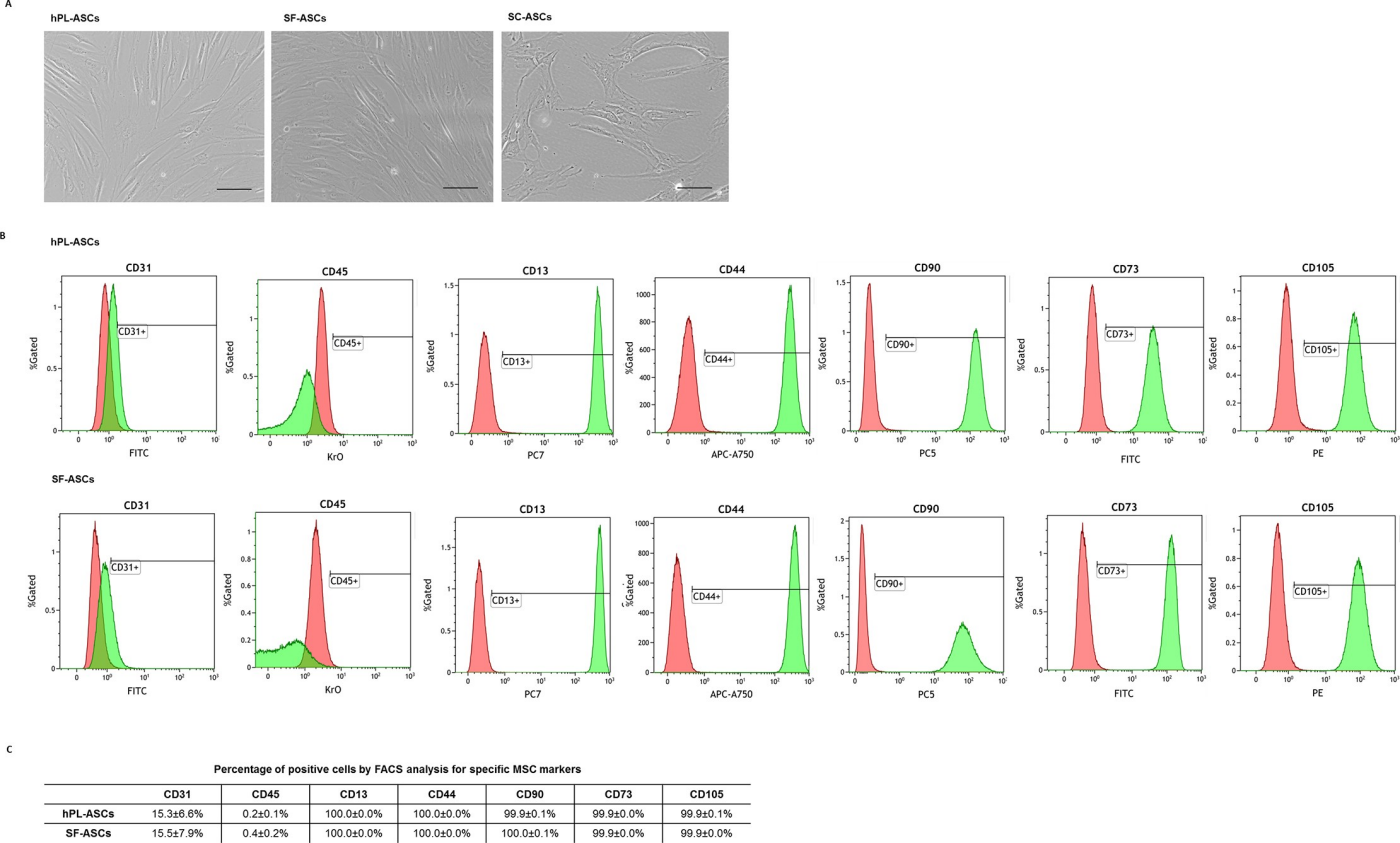
hPL-ASC and SF-ASC appearance and stem cell surface marker patterns of expression. (A) Representative micrographs of ASCs at passage 4 cultured in serum free medium conditions (hPL-ASCs and SF-ASCs) as well as of ASCs cultured in standard laboratory condition (SC-ASCs) using MEM-alpha as growth medium supplemented with 10% of FBS provided by Sigma Aldrich (optical microscopy: 10X; scale bar 200 μm). (B) Representative expression of the typical mesenchymal stem cell surface markers (CD13, CD44, CD90, CD73 and CD105), of the endothelial marker CD31 and of the hematopoietic antigen CD45 for both populations at passage 4 (red: isotypic control, green: ASCs). (C) Quantification of the above depicted markers, pooled for all lines (n = 4).

**Fig 2:**
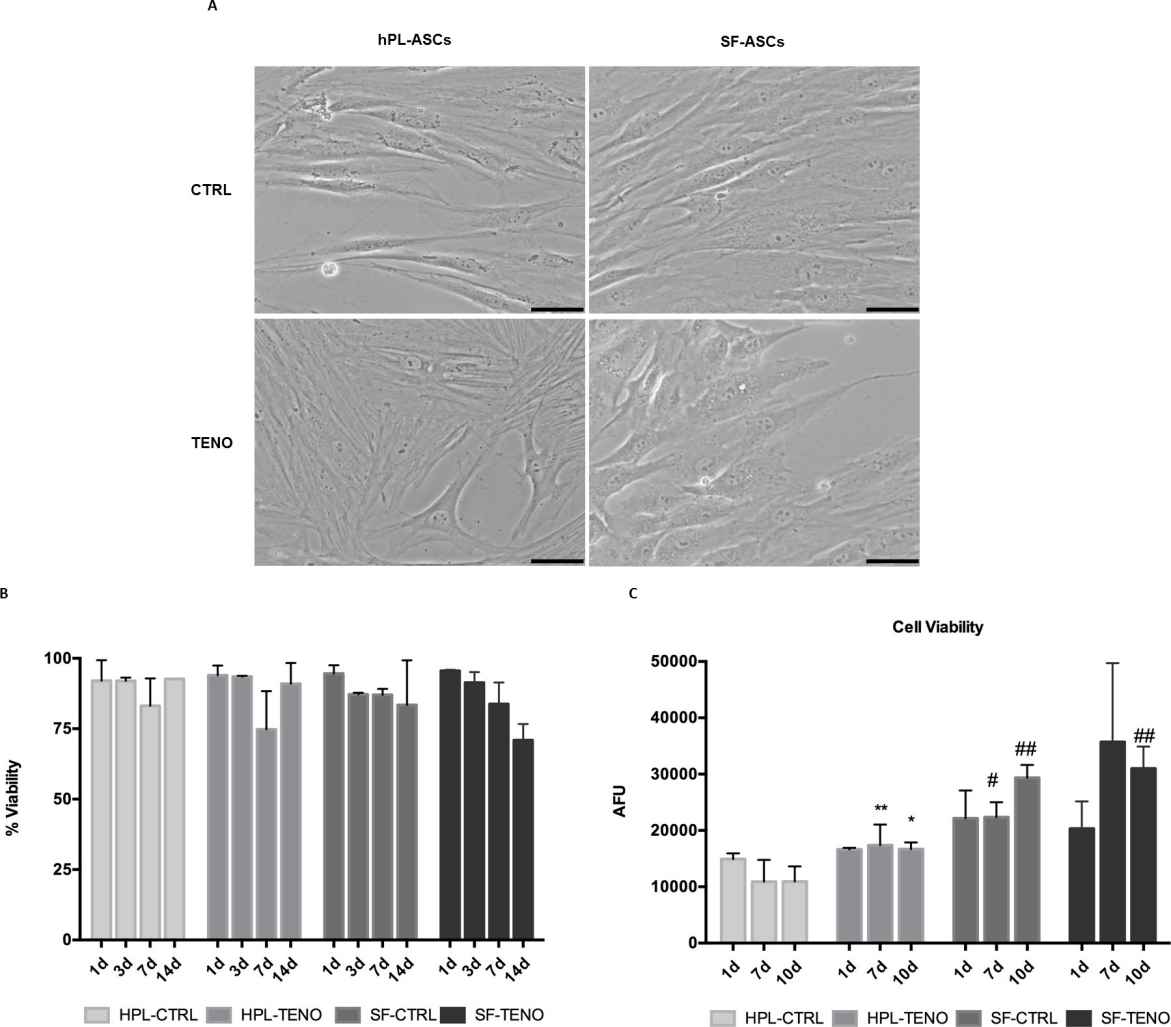
Morphological appearance and cell viability of hPL-ASCs and SF-ASCs during tenogenic induction. (A) Cell morphology of CTRL and TENO hPL-ASCs and SF-ASCs at 3 days of differentiation is shown (optical microscopy 20x; scale bar 200 μm). (B) Percent of viable CTRL and TENO hPL and SF-ASCs at 1, 3, 7 and 14 days (n = 3). Data were expressed as average ± standard deviation of percentage of viable cells. (C) Cell viability of CTRL and TENO hPL and SF-ASCs at 1, 7 and 10 days of differentiation (n = 3). Data were expressed as average ± standard deviation of arbitrary fluorescence units (AFU). * p<0.05, **p<0.01 for TENO versus CTRL cells; # p<0.05, ## p<0–01 for hPL-ASCs vs SF-ASCs.

The average number of ASCs isolated from subcutaneous adipose tissue of 4 female donors was 2.9 ± 1.5 x 10^5^ ASCs per ml of raw adipose tissue. After cell expansion, ASCs cultured in collagen type I coated flasks with hPL or SF media appeared viable and with the typical fibroblastic-like morphology as shows in Fig 1A. Moreover, in order to confirm the maintenance of the MSC nature of cultured ASCs, immunophenotyping was performed in both hPL and SF ASC populations according to the International Society for Cell Therapy (ISCT) standards [33, 34]. Both hPL-ASCs and SF-ASCs were negative (<5%) for the endothelial marker CD31 and the hematopoietic antigen CD45 (Fig 1B and 1C). Concomitantly, more of the 95% of cells were positive for the conventional mesenchymal surface antigens CD105, CD90 and CD73, as well as the stromal markers CD13 and CD44 without any statistical significant differences depending from the culture conditions (Fig 1B and 1C). After tenogenic induction, both hPL-TENO and SF-TENO ASCs appear without signs of suffering and with more rounded morphology and cytoplasmatic content in comparison to the respective undifferentiated control cells, hPL-CTRL and SF-CTRL, as shown in Fig 2A. Moreover, the different culture conditions didn't affect significantly the percentage of viable cells during the time of culture (Fig 2B). Interestingly, the cell viability associated with the metabolic activity of SF-CTRL was higher of what observed in hPL-CTRL cells and resulted in increases of +105% (p<0.05) and +169% (p<0.01) at 7 and 14 days, respectively (Fig 2C). Moreover, the tenogenic media increased again the cell viability in both hPL-TENO (7d: +60%, p<0.01) and SF-TENO (7d: +60%) in comparison to the respective CTRLs, without any significant differences related to the SF media.

**Fig 3:**
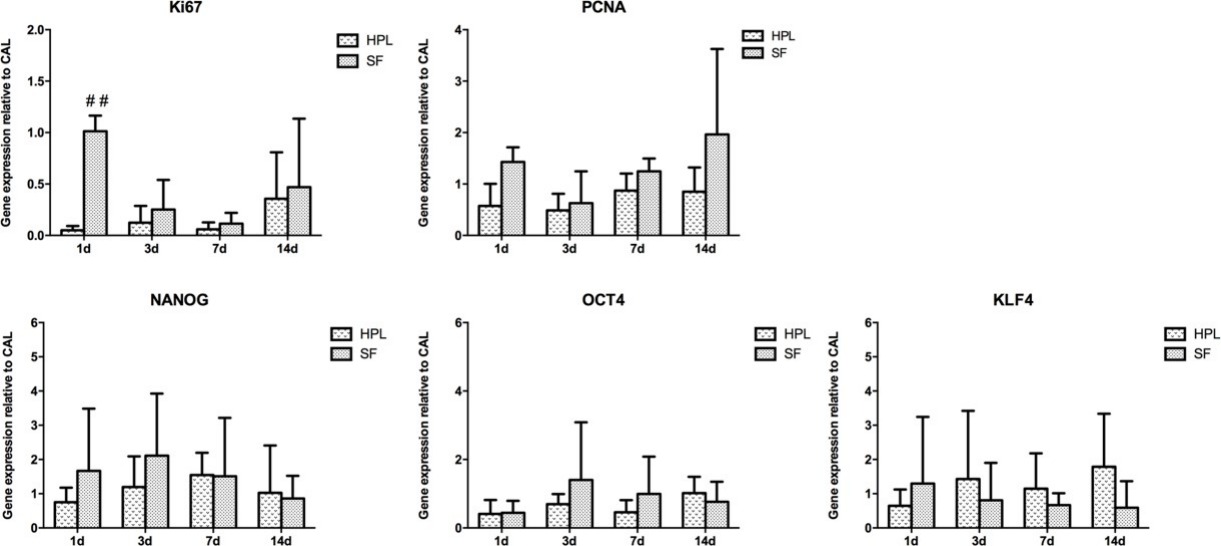
Gene expression of cell proliferation and embryonic stem cell markers in hPL-ASCs and SF-ASCs. Evaluation of Ki67, PCNA, NANOG, OCT4, KLF4 gene expression determined by quantitative real-time PCR in CTRL and TENO hPL and SF-ASCs at 1, 3, 7 and 14 days of culture (n = 4). Data were normalized against the expression of the housekeeping GAPDH, GUSB and YWHAZ genes and expressed as relative to the calibrator (CAL). ## p<0.01 for hPL-ASCs vs SF-ASCs.

**Fig 4:**
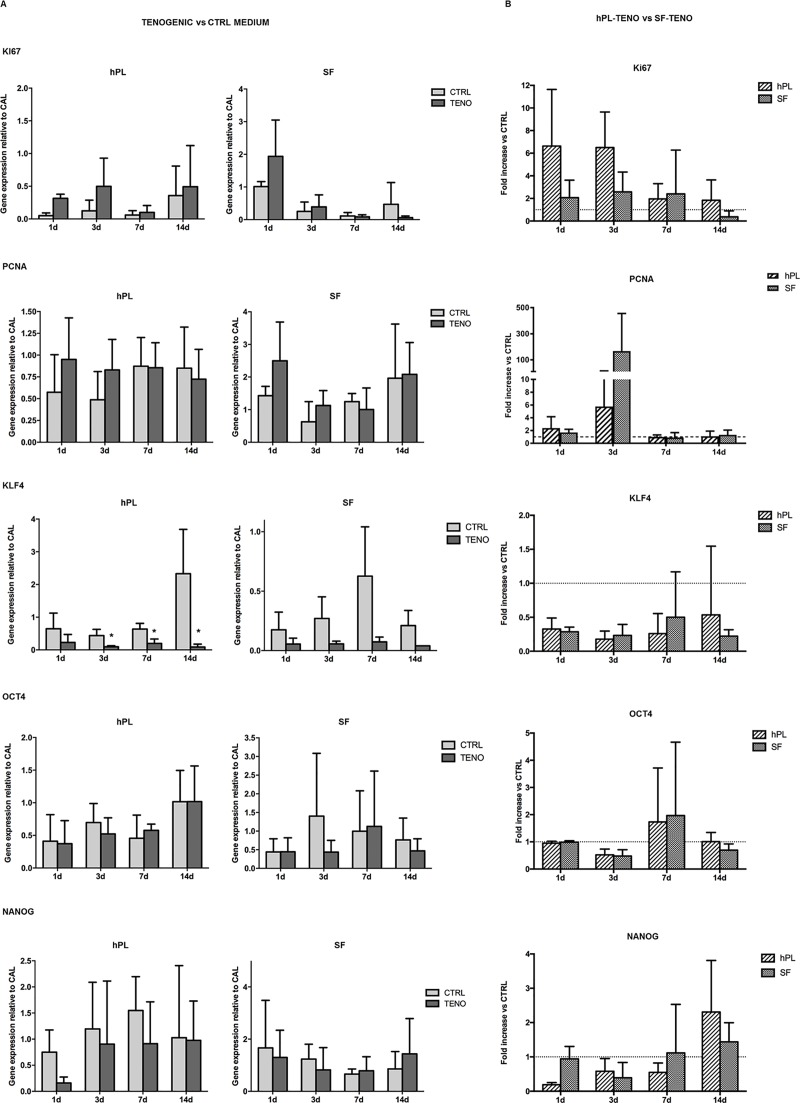
Gene expression of cell proliferation and embryonic stem cell markers after tenogenic differentiation. (A) Effect of tenogenic induction on Ki67, PCNA, OCT4, KLF4 and NANOG gene expression in CTRL and TENO hPL and SF-ASCs at 1, 3, 7 and 14 days of differentiation (n = 4). Data were normalized against the expression of the housekeeping GAPDH, GUSB and YWHAZ genes and expressed as relative to the calibrator (CAL). * p<0.05 for TENO vs CTRL cells. (B) Normalized values of hPL-TENO and SF-TENO to their CTRL. Data expressed as average fold increase ± standard deviation compared with the respective CTRL cells (dashed line means equal).

The expression of markers strictly associated with cell proliferation, Ki67 and proliferating cell nuclear antigen (PCNA) and with the embryonic stem cell markers Kruppel-like factor 4 (KLF4), octamer-binding transcription factor 4 (OCT4) and NANOG, has been also evaluated (Figs 3 and 4). The level of mRNA of Ki67 observed in SF-CTRL ASCs at day 1 was statistically significantly 2-fold increase higher in respect to the hPL-CTRL ASCs (p<0.01, Fig 3). For what concerns the expression of PCNA, NANOG, OCT4 and KLF4 genes no significant differences were observed in both hPL-CTRL and SF-CTRL media at all time points (Fig 3). After tenogenic induction, the expression of both Ki67 and PCNA were up-regulated already at early time points in both hPL-TENO and SF-TENO with respect to the relative CTRLs. Nevertheless these differences are not statistically significant, probably due to the high inter-donor variability (Fig 4A). In particular, differentiated ASCs in hPL media showed the highest levels of Ki67 at 3 days with fold change increases versus CTRL of 6.5 ± 3.1 whilst, at the same time point, the expression of PCNA was higher in TENO-SF ASCs with a fold change increases versus CTRL of 167.3 ± 287.9 (Fig 4B). Concerning the expression of stem cell markers, KLF4 levels were significantly decreased at all time points with the minimum peak observed at 3 days, in both hPL-TENO (3d: -0.94; p<0.05) and SF-TENO ASCs (3d: -0.87) with respect to the respective CTRL cells and without any significant differences between the two xeno-free medium cultures (Fig 4A and 4B). On the other hand, the expression of OCT4 and NANOG was similar in TENO and CTRL cells and without any significant differences between hPL and SF ASCs (Fig 4A and 4B).

### Tenogenic induction in both SF-TENO and hPL-TENO up-regulated SCX, COL1A1, COL3A1, COMP and MMPs tendon-related genes

**Fig 5:**
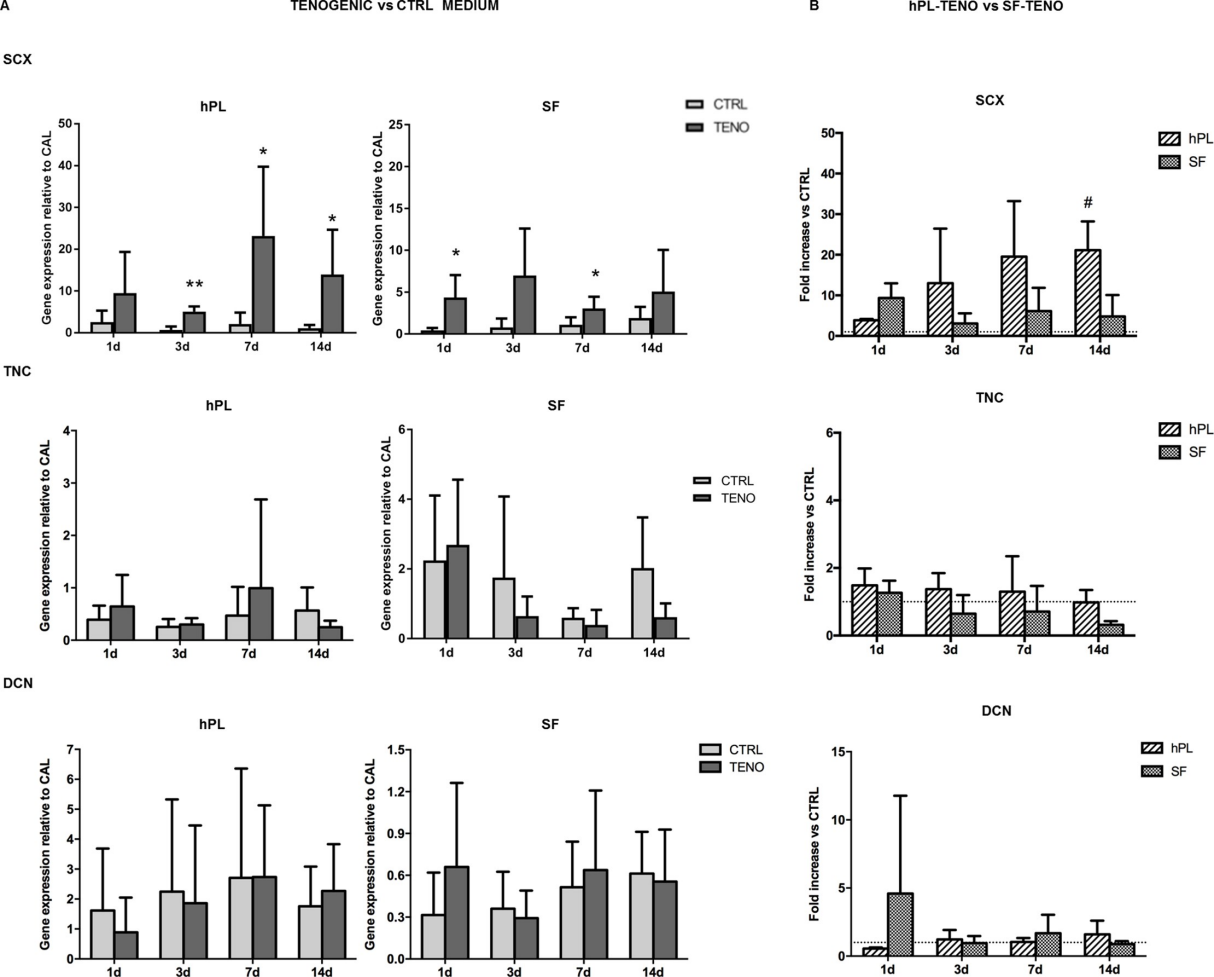
Gene expression of tendon-related markers after tenogenic differentiation. (A) Effect of tenogenic medium on SCX, TNC and DCN gene expression in hPL and SF at 1, 3, 7 and 14 days of differentiation (n = 4). Data were normalized against the expression of the housekeeping GAPDH, GUSB and YWHAZ genes and expressed as relative to the calibrator (CAL). * p<0.05, **p<0.01 for TENO versus CTRL cells. (B) Effect of serum-free media between hPL-TENO and SF-TENO. Data are expressed as average fold increase ± standard deviation compared with the respective CTRL cells (dashed line set at 1). # p<0.05 for hPL versus SF.

**Fig 6:**
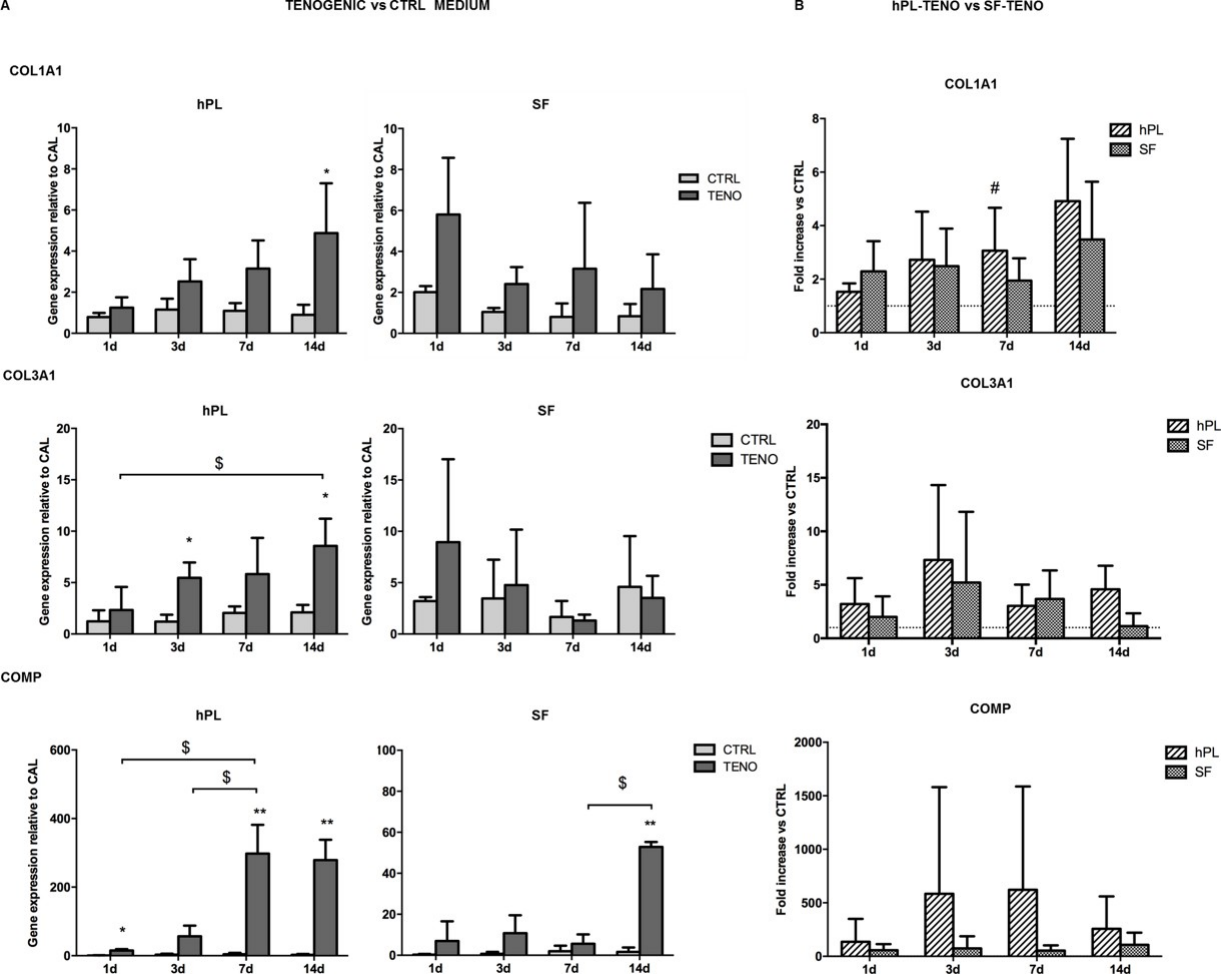
Gene expression of tendon extracellular-related marker after tenogenic differentiation. (A) Effect of tenogenic medium on COL1A1, COL3A1 and COMP gene expression in hPL and SF at 1, 3, 7 and 14 days of differentiation (n = 4). Data were normalized against the expression of the housekeeping GAPDH, GUSB and YWHAZ genes and expressed as relative to the calibrator (CAL). * p<0.05, **p<0.01 for TENO vs CTRL cells; $ p<0.05 for differences between time-points. (B) Effect of serum-free media between hPL-TENO and SF-TENO. Data are expressed as average fold increase ± standard deviation compared with the respective CTRL cells (dashed line set at 1). # p<0.05 for hPL versus SF.

**Fig 7:**
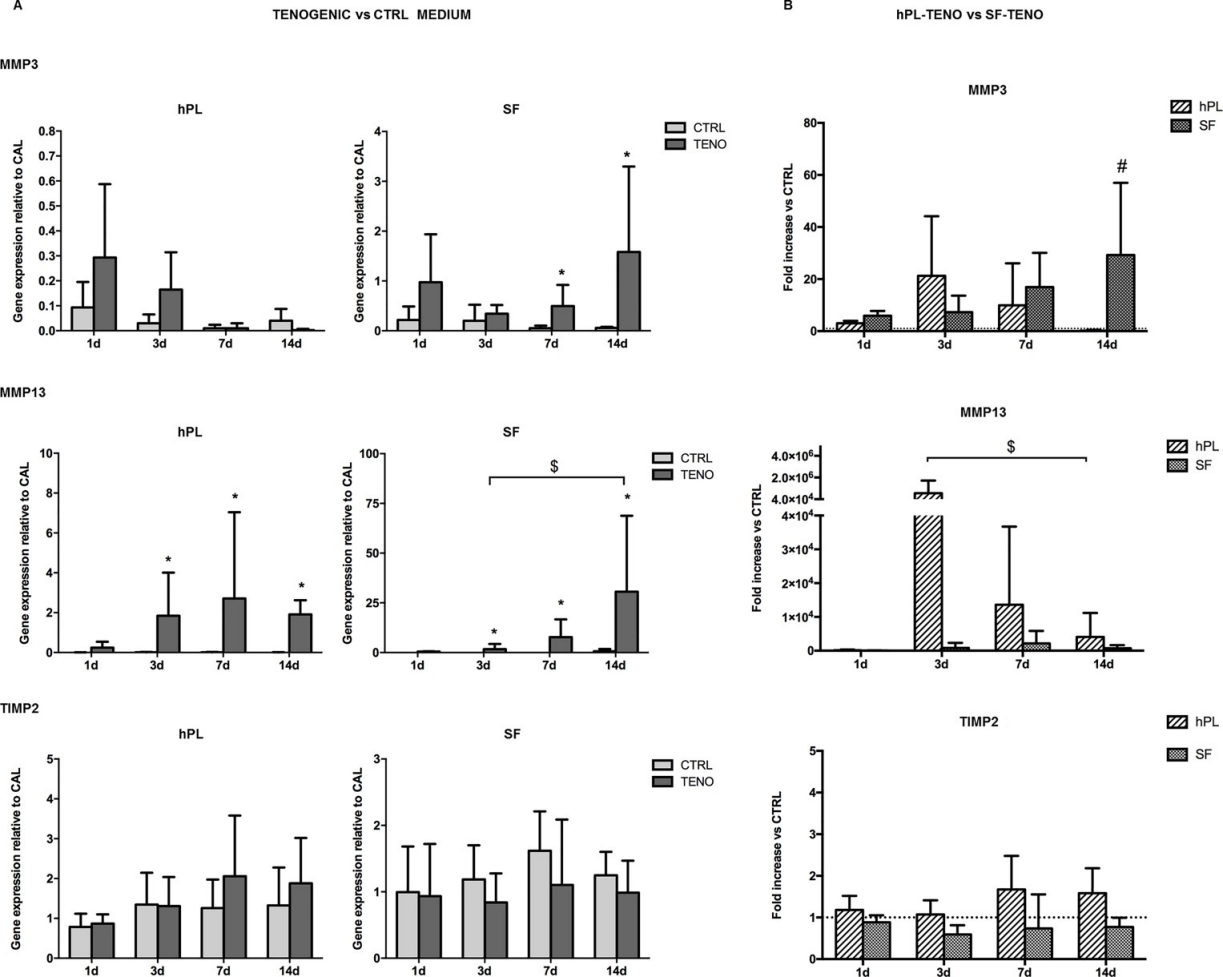
Gene expression of MMP3, MMP13 and TIMP2 after tenogenic differentiation. (A) Effect of tenogenic medium on MMP3, MMP13 and TIMP2 gene expression in hPL and SF at 1, 3, 7 and 14 days of differentiation (n = 4). Data were normalized against the expression of the housekeeping GAPDH, GUSB and YWHAZ genes and expressed as relative to the calibrator (CAL). * p<0.05 for TENO vs CTRL cells; $ p<0.05 for differences between time-points. (B) Effect of serum-free media between hPL-TENO and SF-TENO. Data expressed as average fold increase ± standard deviation compared with the respective CTRL cells (dashed line set at 1). # p<0.05 for hPL versus SF.

ASCs were cultured on pre-coated collagen I culture flasks in hPL or SF media in the presence of the selected soluble factors AA, BMP-12, CTGF and TGF-β3 to verify the efficacy in the induction of tenogenic gene expression and to compare the two *in vitro* systems. Expression of genes known to be involved in the process of tendon development and encoding for ECM proteins were analyzed in all ASC cultures after 1, 3, 7 and 14 days of differentiation induction as reported in Fig 5. The presence of the selected tenogenic soluble factors in both hPL and SF cultures was able to induce the SCX expression, transcription factor a tendon- and ligament-specific, already after 1 day of differentiation, with statistically significant increases of 2.7 and 5.3 times (p<0.05) respectively, in comparison to CTRL cells (Fig 5A). This significant up-regulation was also observed at following time points in both hPL- and SF-TENO ASCs with fold increases of 6.4 and 3.5 at 3 days (p<0.01), of 10.0 and 1.7 at 7 days (p<0.05) and of 11.7 and 1.6 at 14 days (p<0.05), respectively, in comparison with the CTRL cells. In particular, a time-dependent SCX up-regulation was observed in hPL-TENO that at 14 days showed a 21-fold change significantly higher than what observed in SF-TENO at the same time point (p<0.05) (Fig 5B). Concerning the expression of TNC, typically considered a marker of tenogenic differentiation, and DCN, a proteoglycan that stabilizes and aligns collagen type I and III fibrils in tendons, only not significant increase was observed in both TENO media compared to the CTRL (Fig 5A and 5B). On the other hand, the expression of collagen type I and III, encoding for predominant ECM proteins in tendon tissue, were significantly up-regulated after culture in tenogenic media in both hPL and SF conditions (Fig 6A and 6B). In particular, a time-dependent up-regulation of COL1A1 and COL3A1 was observed in hPL-TENO with a maximum peak at 14 days and statistically significant (p<0.05) fold increases of 4.4 and 3.1 (Fig 6A). Again, SF-TENO showed higher increases of these markers already at day 1 (fold increases of 1.9 and 1.8 for COL1A1 and COL3A1, respectively) compared to undifferentiated cells. Then, the COL1A1 over-expression was maintained until 14 days when SF-TENO showed a significant 1-fold increase (p<0.05) relative to CTRL cells. On the other hand, at the same time point, COL3A1 levels resulted similar to what observed in CTRL cells. As shown in Fig 6B, the different behavior in the COL1A1 expression observed in hPL-TENO in respect to SF-TENO was statistically significant at 7 days of tenogenic induction (p<0.05). Tenogenic induction in both xeno-free conditions was able to induce a strong time-dependent up-regulation of the gene encoding for another ECM protein: the cartilage oligomeric matrix protein (COMP). Indeed, starting from 1 day of differentiation, both hPL-TENO and SF-TENO showed a 17.9 (p<0.05) and 24.0-fold increases, respectively, in comparison CTRL cells until reaching at day 14 a 99.0 and 31.8 fold increases respectively (Fig 6A). Moreover, the fold change increases observed at all time points in hPL-TENO seems to be higher with respect to what observed in SF-TENO, even if this difference was not statistically significant (Fig 6B). Finally, the expression of important regulators of ECM remodeling, the matrix metalloproteinases MMP3 and MMP13 and the tissue inhibitor of metalloproteinase TIMP-2, was evaluated in all ASC cultures and shown in Fig 7. After day 1 and 3 of differentiation hPL-TENO showed a 2.1 and 4.4 fold increases, respectively, in respect to CTRL whilst SF-TENO showed fold increases of 8.6 (p<0.05) and 27.6 (p<0.05) at day 7 and 14 in comparison to the respective CTRL cells (Fig 7A). The different timing in the MMP3 expression induction between the two TENO populations was also confirmed at 14 days by a statistical significant difference in SF-TENO that showed higher fold change increases (p<0.05) with respect to what observed in hPL-TENO (Fig 7B). Moreover, a statistically significant over-expression of MMP13 gene was observed starting from 3 days in both hPL-TENO and SF-TENO, in comparison to CTR cells (p<0.05) (Fig 7A). Moreover, hPL-TENO showed higher fold change increases in the MMP13 levels respect to SF-TENO (Fig 7B). For what concern the TIMP2 marker, only hPL-TENO seems to show a slight fold increases at 7 days (0.6) and 14 days (0.4), in comparison to CTRL cells, even if these differences were not statistically significant (Fig 7A and 7B).

### Tenogenic media induced expression of proteins scleraxis, collagen and tenomodulin

**Fig 8:**
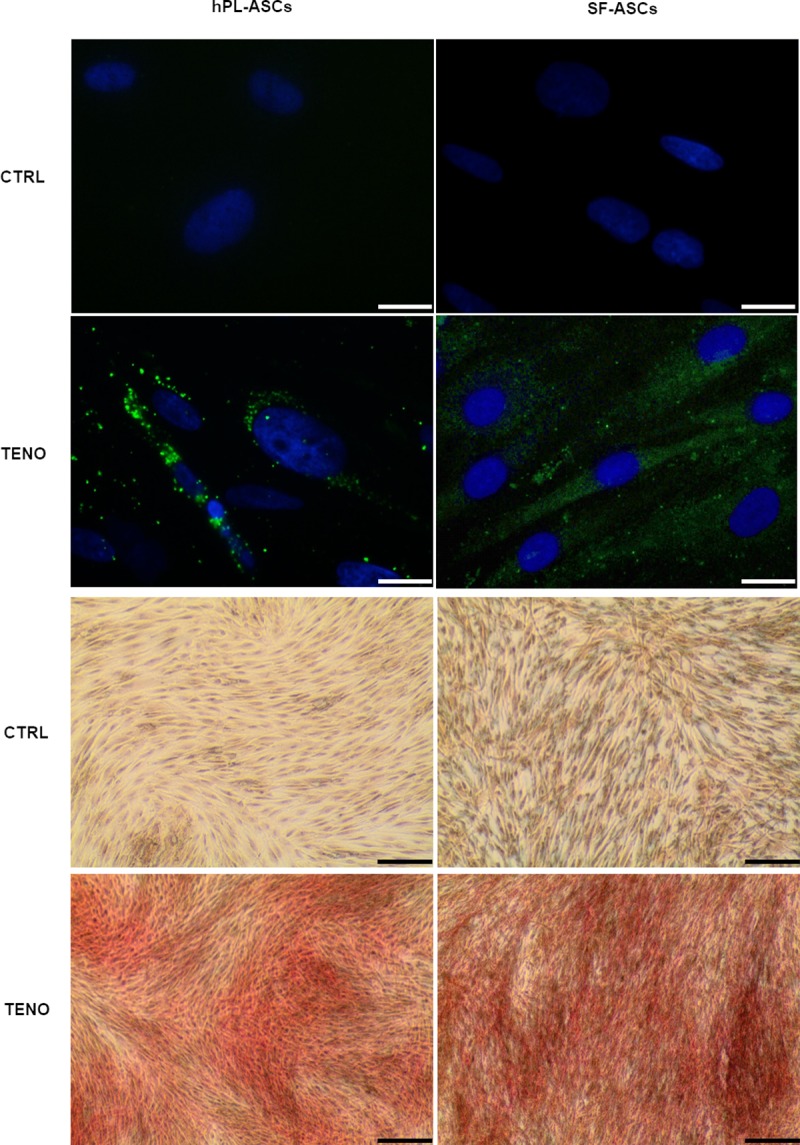
Scleraxis expression and collagen matrix deposition after tenogenic differentiation. Upper four panels show representative images of scleraxis expression (green) in CTRL and TENO cells at 3 days of differentiation (the nuclei were stained with DAPI, blue) captured by fluorescence microscope (40x; scale bar 20 μm); four panels on the button show representative images related to the collagen matrix deposition, stained by Sirius Red (10x; scale bar 100 μm), that occurred after 7 days differentiation in hPL-ASCs and SF-ASCs.

**Fig 9:**
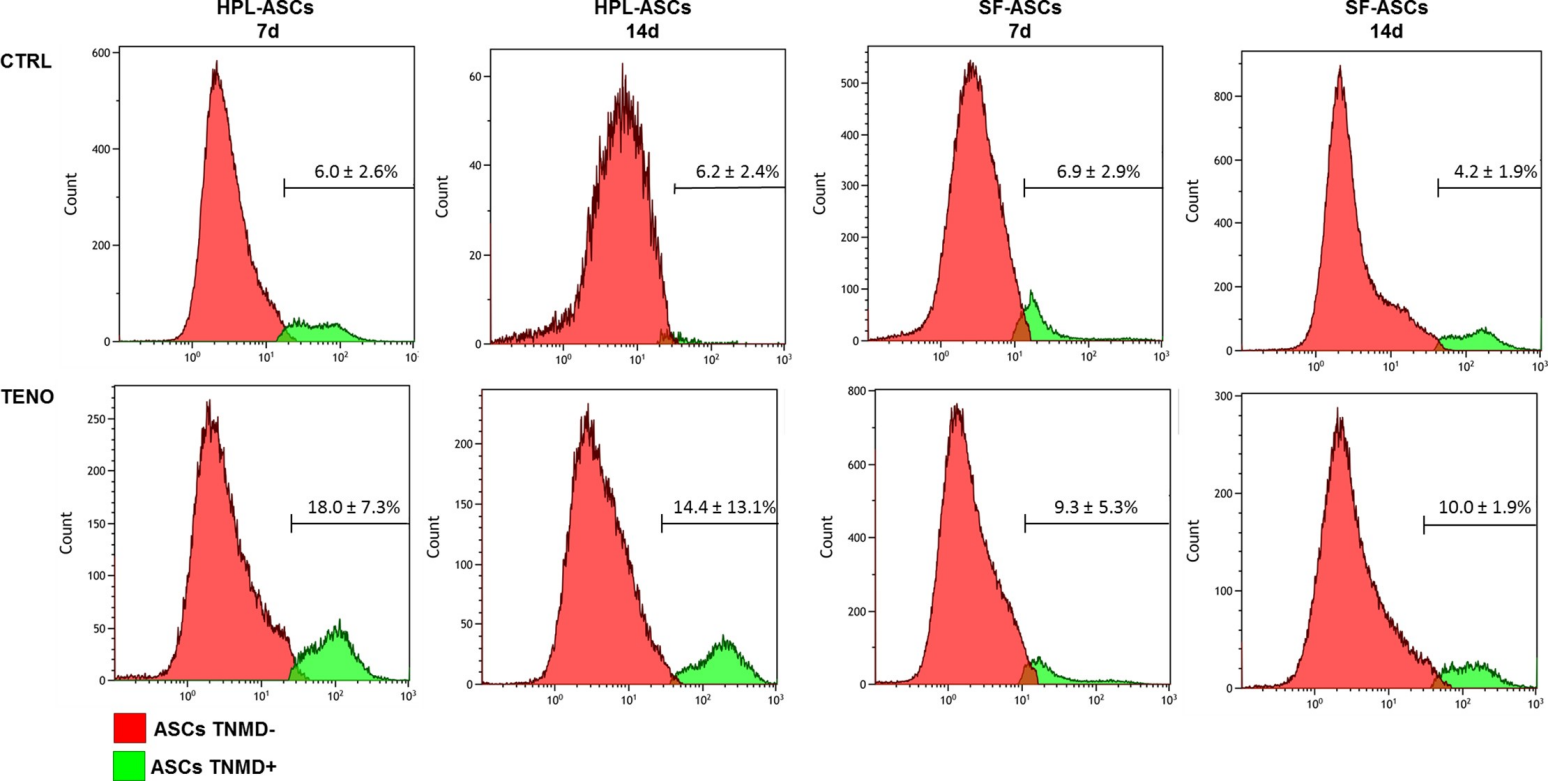
Tenomodulin expression on hPL-ASC and SF-ASC surfaces. Representative histograms of percentage of the subpopulation of hPL-ASCs and SF-ASCs positive (green) and negative (red) for tenomodulin (TNMD) surface expression at 7 and 14 days of culture in CTRL and TENO medium. Data related to three ASC population are expressed as mean ± standard deviation (n = 3). * p<0.05 for TENO versus CTRL cells.

After 3 days of TENO-induction both hPL-ASCs and SF-ASCs expressed scleraxis at protein level as confirmed by immunofluorescence staining showed in Fig 8. A massive collagen matrix deposition was also detected at 7 days of differentiation in both hPL-TENO and SF-TENO (Fig 8). Moreover, the protein expression of the tendon-related marker tenomodulin (TNMD) was evaluated by FACS analysis in hPL-ASCs and SF-ASCs at 7 and 14 days of differentiation (Fig 9). Interestingly a very low percentage of TNMD-positive cells were already present at 7 days in both hPL-CTRL (6.0 ± 2.6%) and SF-CTRL (6.9 ± 2.9%) and they were also observed in similar amount at 14 days. However, the culture with the tenogenic medium was able to induced increases in tenomodulin expression similarly in both hPL-TENO and SF-TENO populations. Indeed, at 7 and 14 days of differentiation, TNMD-positive hPL-TENO cells were 18.0 ± 7.3% and 14.4 ± 13.1%, respectively, with an increase of +66.4% and +57.0% respect to what observed in hPL-CTRL at the same time points. The same trend was observed in SF-TENO that showed significant increases of +26% and +58% (p<0.05) in the expression of tenomodulin at 7 and 14 days of differentiation, respectively, in comparison with SF-CTRL.

## Discussion

Adipose tissue is one of the best and most convenient sources of MSC isolation due to its wide availability as surgical waste material. For this reason, in recent years, several cell and tissue banks have focused their activity on adipose tissue preservation and of its related ASCs population [8]. Moreover, encouraging pre-clinical evidences showed that ASCs could improve tendon healing with a reduction in the inflammatory response and improving fiber arrangement and tendon organization [34–36].

The main goal of the present study was to evaluate the tenogenic differentiation of ASCs in xenogenic and/or serum-free medium conditions not only to clarify some of the events implied in tendon regeneration but also to contribute in defining the best culture environment for cell-based therapy approaches. Previous studies showed that ASCs cultured in SF medium, differently than serum containing media, still maintain the multi-differentiation potential toward adipogenic, osteogenic and chondrogenic lineages and surface marker expression profile characteristic of MSCs [37, 38]. In agreement with these reports and with the minimal criteria for defining multipotent MSCs, here we have demonstrated that ASCs plated in coated collagen type I flasks and cultured in a unique xenogenic and serum-free culture medium (SF) or in hPL supplemented medium maintained the peculiar features of progenitor cells including the typical fibroblastic spindle-like morphology and the stem cell markers expression [33]. Indeed, both hPL-ASCs and SF-ASCs showed the specific MSC immunophenotype profile and expressed the transcription factors which are essential for self-renewal maintenance and pluripotency in embryonic stem cells such as KLF4, OCT4 and NANOG [33, 39]. Surprisingly, cells cultured in SF-CTRL were characterized by higher metabolic activity and proliferation ability in respect to hPL control cells. Gene expression of the widely used markers of cell proliferation, Ki67 and proliferating cell nuclear antigen (PCNA, was also performed. We observed in all samples an increased expression of these markers for 14 days even if cells cultured in SF showed an increase of 2-fold (p<0.01) only at early time points compared with cells cultured in hPL medium. Moreover, cells cultured in SF had the highest levels of metabolic activity in respect to hPL-ASCs without significant differences between CTRL and TENO. Indeed, the tenogenic medium was able to induce a marked increase of metabolic activity only in hPL-ASCs in respect to hPL-CTRL. From these data we demonstrated first the feasibility of our chemically defined SF medium to sustain ASCs growth in culture and to maintain their MSCs characteristics in vitro and that our tenogenic medium not negatively affects ASCs viability during the time of differentiation in vitro.

To the best of our knowledge, this is the first study that compares the tenogenic differentiation potential of human ASCs in a unique xenogeneic and serum-free culture media supplemented with a mixture of ascorbic acid, BMP-12, CTGF and TGF-β3 because their already know to exert a role in tendon development and repair. The tenogenic differentiation of SF-ASCs and hPL-ASCs was here evaluated in the first instance by monitoring the expression of a panel of genes related to tendon development pathway (e.g. SCX), tendon and extracellular matrix (ECM) related genes (e.g. DCN, TNC, COL1A1, COL3A1, COMP, the metalloproteinase MMP-3 and MMP-13 and tissue inhibitor protein TIMP-2) and then by analyzing the protein expression of scleraxis, collagen-matrix and tenomodulin.

The dry weight of normal tendons consists in ECM composed mainly of collagen type I and III, and in minor percentage of elastin embedded in a proteoglycan-water matrix, proteoglycans, glycosaminoglycans and structural glycoproteins [40]. The cellular component is present in very low percentage and consists in terminally differentiated cells named tenocytes that, together with a small niche of tendon-derived stem cells, are responsible for maintaining ECM homeostasis and collagen molecules synthesis [8, 41]. Moreover, matrix metalloproteinases (MMPs) are important regulator of ECM remodeling and include secreted collagenases (i.e MMP13) and stromelysins (i.e MMP-3) among others, whose enzymatic activity is balanced by a family of tissue inhibitors, the tissue inhibitors of metalloproteinases (TIMPs) [1, 2]. The ECM microenvironment is essential for stem cells maintenance as well as to normal tissue development and maintenance. The connective tissue growth factor (CTGF) is a cysteine-rich protein growth factor highly expressed at the early stage of tendon repair since their mRNA expression was found to be increased in a chicken tendon injury model and the MSC treatment with this GF promoted the collagen type I and tenascin-C expression [42, 43]. The TGF-β signaling plays a major role as a potent scleraxis (SCX) tendon marker gene expression and collagen production inducer during tendon formation also observed in *in vitro* differentiated equine embryo-derived stem cell cultures [44–47]. Furthermore, the BMPs family, including BMP-12, has been shown to induce formation of new connective tissue and to enhance tendon repair in several tendon injury models [48–50]. The presence of BMP12 in the culture medium of both canine and human ASCs effectively increased the SCX expression at both mRNA and protein level [19, 8]. A stimulatory effect of CTGF and BMP12 has been suggested by Liu and colleagues during tenocyte lineage differentiation markers through a direct physical interaction of these GFs [51]. Other studies reported that, in combination with BMP-12 or CTGF growth factor, ascorbic acid, an essential factor acting in the cross-linking catalysis of collagen during collagen fibril formation in ECM during tendon development, was able to enhance the expression of SCX and collagen type I and III on human ASCs and tendon stem/progenitor cell (TSPCs) culture [17, 18, 52].

Our results demonstrated that the tenogenic medium induced significant positive increases of gene expression of the transcription factor scleraxis already after 1 day as well as of the others tendon-related markers COL1A1, COL3A1, COMP, MMP3 and MMP13 in both hPL-ASCs and SF-ASCs. These data were also confirmed at protein level expression as revealed by immunofluorescence analysis of scleraxis and by total collagen production staining. However, the differentiation of ASCs in hPL medium was more able to positively regulate the expression of these tendon-related markers in comparison to what observed in SF-TENO. Since it is know that during tendon development, scleraxis expression is associated with formation of tendon/ligament primordium whereas tenomodulin expression increases markedly in parallel with maturation of tendons and ligaments, we here have been also evaluated its expression on ASC surface [53, 54]. Tenomodulin, is a type II transmembrane glycoprotein and its expression in mature tenocytes has been implicated in regulating their proliferation and matrix organization [55]. Recently, it has been also demonstrated that the overexpression of tenomodulin in murine MSCs significantly enhanced cell proliferation, gene expressions of tendon-related markers and promoted neotendon-like tissue formation in vivo indicating a positive regulation of MSC tenogenic differentiation [56]. In according to this, analysing the tenomodulin expression by cytofluorimetric analysis, we identify a 2-fold increase in percentage of TNMD-positive cells in differentiated ASCs respect to control at 14 days. Only ASCs in hPL-TENO medium showed a 3-fold increase of this marker already after 7 days of differentiation. Interestingly, undifferentiated ASCs were positive for this marker in low percentage and without differences depending on time of culture and type of serum free medium used. Another key ECM component is representing by COMP since it is able to enhance the kinetics of collagen fibrillogenesis and is correlated with healthy tendon healing *in vivo* [57, 58]; thus the up-regulation of COMP observed only in the TENO group is potentially of clinical relevance and benefit. Concerning the expression of genes TNC and DCN, typically ECM protein in tendon, slight increases were observed in TENO ASCs populations. In a previous study on human ASCs, Goncalves et colleagues showed high expression of tenascin C and decorin only after 21 days in culture with EGF and bFGF supplemented media [59]. These results could suggest that 14 days of differentiation is not sufficient to elicit a strong expression of these markers that could also be driven by other GFs not included in ours tenogenic media. On the other hand, interestingly, we observed that the gene expression of other ECM components such as the matrix remodeling proteins MMP-3 and MMP-13 was higher in differentiated cells of both ASC group whilst any mRNA levels of these markers was observed in the respective CTRL cells. The balance between the MMPs and their inhibitors, the TIMPs, determines the composition of the ECM and thereby helps to control tendon generation and function. It is known that MMP-3 activity can degrade a broad range of target endopeptides as well as activate other MMPs, while MMP-13 is specific for collagens. However, TIMP-2 is a nonspecific inhibitor of various MMPs and we found no significant changes in the gene expression levels after tenogenic induction in both ASC groups. These findings suggest that GFs used here play a role in both normal turnover and maintenance of the tendon like cells and in the connective tissue degradation process associated with tendon healing.

Altogether, these data confirm that the combination of AA, TGF-β3, BMP-12 and CTGF efficiently drive the tenogenic differentiation of ASCs suggesting their profound role in tendon development and repair process. Moreover, although both hPL and Sf medium well sustain cell growth in vitro, hPL medium seems to be more performing to address ASC differentiation toward tendon in respect to SF medium as relieved by the highest levels of tendon-related marker expression observed. Beside these observations, some explanations about the statistical significant differences observed comparing the two differentiation media (SF-TENO versus hPL-TENO), could be caused by differences in doses of growth factors due to the not strictly chemically defined and standardized nature of hPL medium together with a certain intrinsic lot-to-lot variability. One limitation of this study is caused by the high inter-donor variability that often represents a critical point when dealing with primary cells as well as the isolation and extensive culture *in vitro* of ASCs using SF medium. Moreover, since tendon is a mechano-responsive tissue, suitable markers could be also further investigated in hPL-TENO and SF-TENO in both static and dynamic *in vitro* conditions to better explain the ASCs role in tendon healing processes as well as the use of tissue-engineered three-dimensional scaffolds for the tremendous capacity to closely mimic in vivo cellular environments.

## Conclusion

Cell-based therapies using ASCs with their multiple properties are constantly increasing to manage numerous orthopedic problems including tendon injuries. For the cell therapies development, safety, efficacy, reproducibility and quality are highly prioritized. With a clinical-grade medium formulation, the safety and quality of the transplanted stem cells may be significantly enhanced. Furthermore a fully defined serum free formulation is a step closer to the development and approval of clinical cell-therapy applications. The GMP compliant approach used in this study, is based on the development of xenogenic and serum-free medium supplemented with a combination of growth factors able to induce the tenogenic differentiation of ASCs in vitro. We demonstrated that our chemically defined SF medium can be used to maintain viability and MSC features of ASCs in culture until their implantation in vivo. Moreover, the supplementation with AA, TGF-β3, BMP-12 and CTGF soluble factors induce ASCs to express a tenocyte-like phenotype providing insights of the earliest events of tendon development and suggesting possible GMP-compliant approaches needed for cell-therapy application.
